# Modular Control of PEG‐Poly(2‐Oxazoline) Co‐Assembly Enables Tunable Aggregate Properties for Intraocular Pressure Management

**DOI:** 10.1002/adhm.202504206

**Published:** 2026-04-09

**Authors:** Yuji Tanaka, Shohei Morikawa, Shigehito Osawa

**Affiliations:** ^1^ Age‐Adaptive Project for Centenarian Mobility, New Industry Creation Hatchery Center (NICHe) Tohoku University Sendai Miyagi Japan; ^2^ Innovation Center of NanoMedicine (iCONM) Kawasaki Institute of Industrial Promotion Kawasaki Kanagawa Japan; ^3^ Department of Ophthalmology, Faculty of Medicine University of Tsukuba Tsukuba Ibaraki Japan; ^4^ Department of Biomedical Engineering Faculty of Life Science Toyo University Asaka Saitama Japan

**Keywords:** biomedical applications, hypotony, intraocular pressure, ophthalmology, polymers

## Abstract

Poly(2‐oxazoline) (POx) polymers are hydrophilic and biocompatible materials with tunable physicochemical properties for biomedical use. Postoperative hypotony, which currently lacks approved pharmacological or device‐based treatment, requires minimally invasive and controllable methods for temporary intraocular pressure restoration. Herein, we present a modular strategy to control POx aggregation via thermally triggered co‐assembly with thermoswitchable poly(2‐n‐propyl‐2‐oxazoline) (PnPrOx) and hydrophilic PEG‐modified PnPrOx (PnPrOx‐PEG). These mixtures remain dissolved at low temperatures but aggregate at physiological temperatures because of the thermo‐switchable behavior of the PnPrOx segments, with PEG content modulating particle size, dispersibility, and fusion. Based on in vivo compositional screening in rabbits, we highlight a 98:2 (w/w) PnPrOx:PnPrOx‐PEG formulation (POx2P) that induces transient intraocular pressure elevation without persistent turbidity or deposition. In a trabeculectomy‐induced hypotony model, POx2P restored intraocular pressure without adverse effects. Microscopy and in vivo imaging suggest that this formulation forms flow‐responsive aggregates that transiently occlude aqueous outflow pathways. We established a compositionally tunable and injectable POx platform for in situ aggregation and pressure modulation, offering a clinically relevant approach for localised intraocular therapy.

## Graphical abstract

A thermo‐responsive poly(2‐oxazoline)‐based injectable formulation is developed to modulate intraocular pressure (IOP) in both normal and hypotonic eyes. By adjusting the PEG content, the aggregation behavior is finely tuned, enabling IOP elevation without long‐term deposition. This strategy offers a minimally invasive approach for managing low IOP while maintaining ocular transparency.

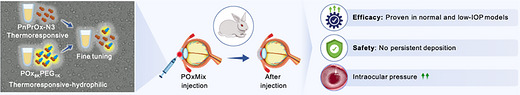

## 1 Introduction

Thermally responsive injectable biomaterials that change structure under physiological conditions are promising tools for localized therapeutic modulation [1, 2, 3]. Examples include thermo‐gelling hydrogels for cancer therapy [3, 4], temperature‐sensitive formulations for arthritis [5, 6], and stimuli‐responsive systems for ocular drug delivery [7]. Spatiotemporal control over material localization is key to enhancing efficacy while minimizing systemic exposure [1, 3, 8]. However, achieving both targeted delivery and functional performance in vivo often requires extensive formulation screening.

Recently, multifunctional biomaterials such as gelatine‐, collagen‐, and zein‐based platforms have demonstrated advanced control over drug delivery and biological interactions [9, 10, 11]. Although these materials highlight the potential of tunable supramolecular and hydrogel systems, they also illustrate a central challenge: identifying therapeutically effective formulations often requires extensive optimization and iterative screening. This process can be time‐consuming and makes it difficult to establish a full design–function relationship within a single study.

Such control is particularly critical in the anterior chamber of the eye, where intraocular pressure (IOP) is tightly regulated by a dynamic balance between aqueous humor production and drainage [12, 13]. Hypotony can occur after both trabeculectomy (TL) and vitrectomy and sustained hypotony can lead to sight‐threatening complications, including choroidal detachment and hypotony maculopathy; therefore, timely restoration of physiologic IOP is clinically essential.

Intracameral viscoelastic hyaluronic acid (HA) is frequently used as a supportive intervention to temporarily increase outflow resistance. A single injection can often restore anterior chamber depth and promote the recovery of aqueous production; however, multiple clinical reports have indicated that HA does not adequately normalize IOP in a subset of patients [14, 15, 16]. Importantly, no standardized or approved pharmacological treatment exists for postoperative hypotony, highlighting the need for alternative approaches with distinct mechanisms of action.

Collagen‐based thermosensitive gels (e.g., atelocollagen punctal plugs [17]) are approved medical devices that occlude tear drainage by gelating at body temperature after injection into the punctum, thereby increasing the tear volume. However, they are unsuitable for aqueous humor modulation, which requires intraocular injection and circumferential delivery along the iridocorneal angle. In addition, these gels often become opaque at body temperature, limiting their use in the anterior chamber, where optical clarity is critical.

Poly(2‐oxazoline) (POx) polymers have emerged as versatile candidates for biomedical applications, owing to their hydrophilicity, biocompatibility, and tunable physicochemical properties [18, 19, 20]. Structural modifications in their side chains or terminal groups enable the precise control of hydrophilicity, thermo‐responsiveness, and interfacial behavior [21, 22], enabling their use in drug delivery [23, 24, 25, 26, 27, 28], and even in clinically evaluated haemostatic devices [29].

For intraocular use, however, the material must accumulate near aqueous outflow sites, transiently obstruct drainage, and maintain transparency—requirements that are difficult to achieve simultaneously. Accordingly, a transient IOP‐elevating injectable material remains an unmet clinical need. Previous strategies often relied on labor‐intensive synthesis and iterative screening of large polymer libraries, hindering translational potential.

To overcome these challenges, we devised a minimalist co‐assembly strategy using just two pre‐synthesized POx‐based components: a hydrophobic homopolymer, poly(2‐n‐propyl‐2‐oxazoline) (PnPrOx), and an amphiphilic POx–PEG (polyethylene glycol) block copolymer (PnPrOxPEG). This modular approach enables the compositional tuning of injectable formulations capable of modulating aqueous outflow and IOP. The aim of the present study was to evaluate the feasibility and therapeutic potential of this system in a translational ocular model.

## 2 Results

### 2.1 Synthesis and Basic Characterization of PnPrOx and PnPrOxPEG

PnPrOx (Figure 1a (i)) was synthesized via the ring‐opening polymerization of 2‐n‐propyl‐2‐oxazoline (nPrOx) using p‐toluene sulphonic acid as an initiator, followed by termination using sodium azide, as outlined in a previous report [30] (Figure S1a). The average molecular weight of PnPrOx was 8 KDa, as determined via 1H NMR spectroscopy. Subsequently, PnPrOx was modified via click chemistry [31, 32] using DBCO‐PEG (Mn = 1 K) (Figure S1b), which acted as a hydrophilic segment to impede the progress of polymer aggregation. According to the 1H NMR spectra and size exclusion chromatograph, PnPrOx was modified to PnPrOx‐b‐PEG in this polymer sample, termed PnPrOxPEG (Figure 1a (ii); Figures S2 and S3).

FIGURE 1Overview of the preparation and time‐dependent microscopic characterization of the PnPrOx/PnPrOxPEG mixture solutions, a series of aqueous mixtures of n‐propylpolyoxazoline (PnPrOx‐N_3_) and polyethylene glycol‐modified polyoxazoline (POx_8K_PEG_1K‐XX_) with varying mixing ratios. These solutions were prepared to investigate their physical properties and behavior under different conditions. (a) Chemical structures of PnPrOx (i) and PnPrOxPEG (ii). (b) Thermo‐responsivity indicates light transmittance in aqueous solutions of PnPrOx (i) and PnPrOxPEG (ii). (c) Schematic illustration of the preparation of the PnPrOx/ PnPrOxPEG mixtures. (d) Macroscopic photograph of PnPrOx/PnPrOxPEG mixtures prepared at ratios of 100/0, 99/1, 98/2, 95/5, 90/10, and 0/100 (w/w) in plastic tubes at 37°C. (e) Typical microscopic images of PnPrOx/PnPrOxPEG mixtures prepared at ratios of 100/0, 99/1, 98/2, 95/5, 90/10, and 0/100 and incubated in a temperature‐controlled chamber at 37°C for at least 5 min. Under these conditions, all formed particles settle and stabilize at the bottom of the glass cell. (f) Phase‐contrast images of various PnPrOx/PnPrOxPEG mixtures prepared at ratios of 100/0, 99/1, 98/2, 95/5, 90/10, and 0/100 and captured at 0 min, 3 min, 3 min + 5 s, 5 min, and 5 min + 5 s after cooling. Particles that did not move at 5 s after being observed at 3 or 5 min after cooling were marked with red circles to indicate adsorption onto the cell surface, whereas unmarked particles remained suspended. (g) Classification of PnPrOx/PnPrOxPEG mixture particles precipitated at 37°C, 3 or 5 min after cooling, categorized as adsorbed onto the cell surface or suspended in the liquid, along with particle counts. (h) Classification and area analysis of PnPrOx/PnPrOxPEG mixture particles precipitated 3 or 5 min after cooling, distinguishing between adsorbed and suspended particles. (i) Number of particles in the area measuring 50 µm × 50 µm, (ii) average area of particles. (i) Schematic depicting the molecular mechanism associated with PnPrOx/PnPrOxPEG precipitation in relation to PnPrOx/PnPrOxPEG mixing ratios.

**Figure:**
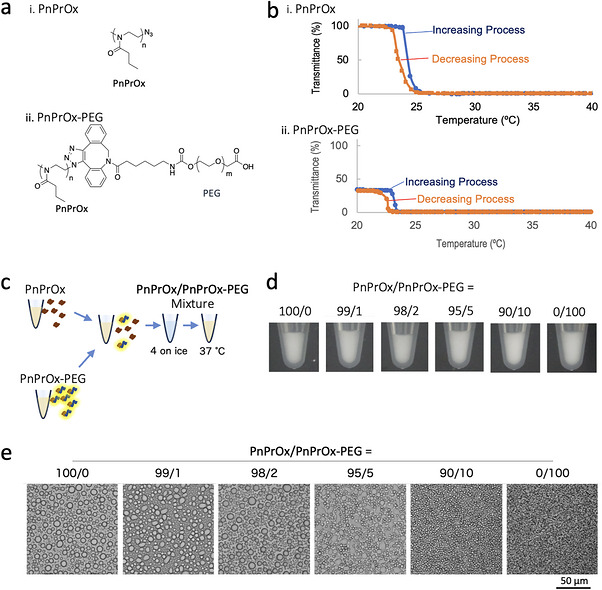

**Figure:**
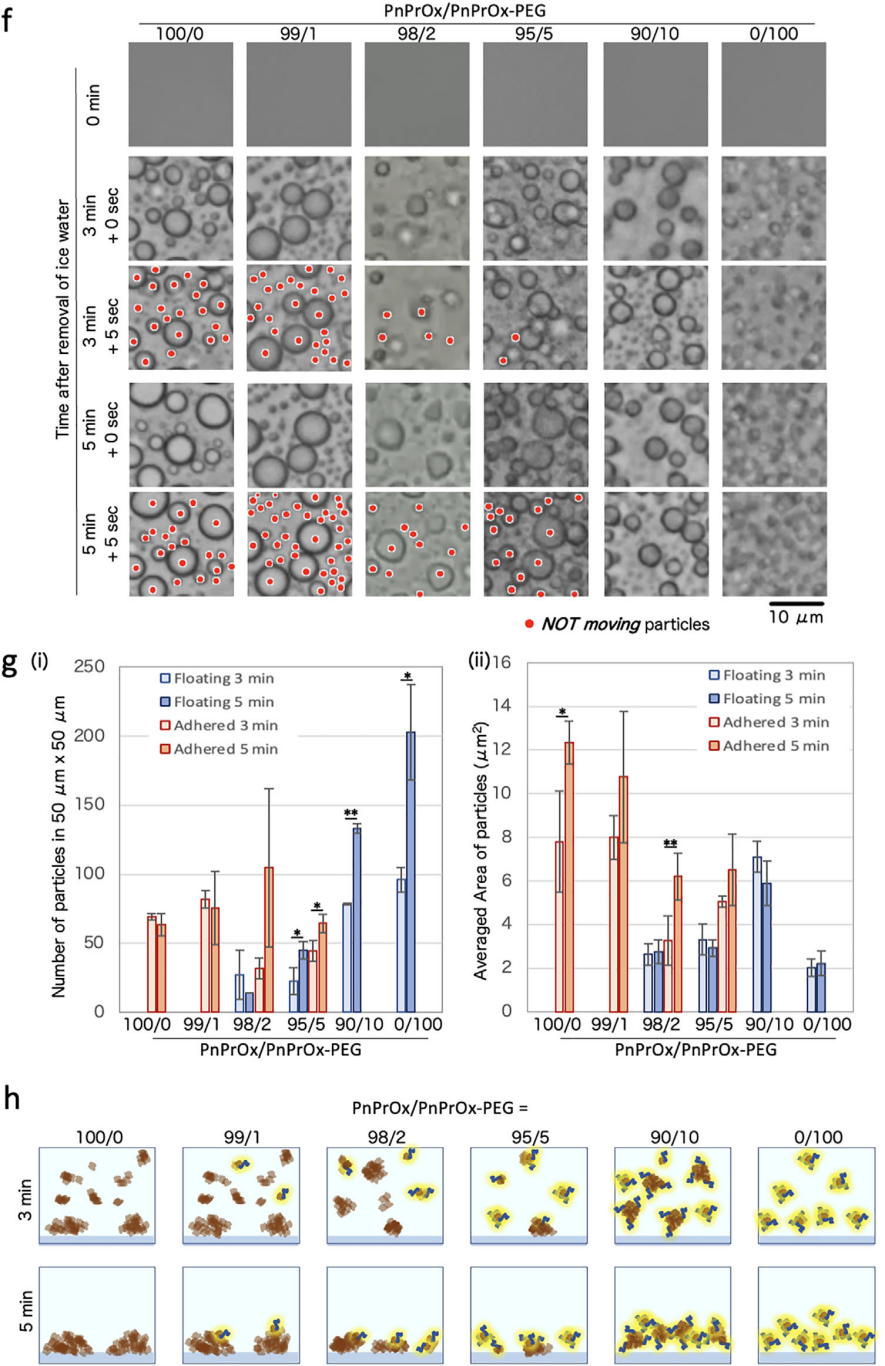

The transmittance of PnPrOx (Figure 1a) and PnPrOxPEG (Figure 1b) (prepared in phosphate‐buffered saline (PBS) at a concentration of 1 mg/mL) exhibited a pronounced and reversible change at approximately 23°C–25°C, decreasing at higher temperatures and increasing at lower temperatures. These findings suggest that the polymers would be assembled or aggregated based on the hydrophobic interaction of the PnPrOx segments.

### 2.2 Time‐Lapse, Phase‐Contrast Microscopy of Thermally Induced Aggregation of Various PnPrOx and PnPrOxPEG Mixtures at 37°C

To examine the effect of PEGylation on PnPrOx segment aggregation, mixtures of PnPrOx‐ and PnPrOxPEG were prepared at varying ratios (Figure 1c). Although all formulations appeared turbid at 37°C, no visible differences in macroscopic appearance were observed (Figure 1d). To further characterize the thermally induced aggregation behavior, time‐lapse phase‐contrast microscopy was performed (Movies S1–S6). All particles settled on the glass surface within 5 min after cooling, with no further movement observed, confirming the completion of aggregation during this timeframe. The supplementary videos confirm that POx2P and other mixing ratios exhibit reversible, temperature‐dependent aggregation behavior consistent with the thermo‐responsive transitions of the individual components shown in Figure 1. Thus, the thermo‐responsiveness of the mixtures themselves was experimentally demonstrated, complementing the transmittance data obtained for the single polymer components. The results showed that a higher fraction of PnPrOxPEG resulted in the formation of smaller, more dispersed particles (Figure 1e). Notably, dynamic Brownian motion was observed exclusively in mixtures with high PnPrOxPEG content.

To elucidate aggregation dynamics, images captured 3 and 5 min after mixing were analyzed. At PnPrOx/PnPrOxPEG ratios of 100/0 and 99/1 (w/w), all particles adhered to the glass substrate. In contrast, at 98/2 and 95/5 (w/w), both adhered and suspended particles were present, with suspended particles exhibiting Brownian motion. At higher PEG fractions (90/10 and 0/100, w/w), nearly all particles remained suspended (Figure 1f,g). Additionally, at 100/0 and 99/1 ratios, the adhered aggregates increased in size over time, whereas at 90/10 and 0/100, the number of floating particles increased without significant changes in size. The 95/5 mixture exhibited intermediate behavior, with both adhered and floating particles increasing in number while maintaining a constant size. At 98/2, the sizes of the adhered particles increased from approximately 2.5 µm^2^ at 3 min to 6.0 µm^2^ at 5 min. These findings suggest that PnPrOx domains promote adhesion and fusion, whereas the PEG segments modulate these interactions. The observed aggregation profiles are presented in Figure 1h. As PnPrOx‐based aggregates are denser than the surrounding buffer, they sediment toward the bottom surface after formation. All microscopic observations were therefore conducted with the focal plane positioned at the chamber bottom, where air bubbles, being buoyant, were absent. Thus, the structures shown in Figure 1f are polymer aggregates rather than air bubbles.

These in vitro aggregation modes (adhesion, fusion, and dispersed suspension) suggested that formulations forming larger or more adhesive aggregates might persist after injection, whereas more uniformly suspended particles may clear more efficiently in vivo.

### 2.3 Screening of PnPrOx/PnPrOxPEG Mixtures for Identifying IOP‐Elevating Formulations in Rabbits

**FIGURE 2:**
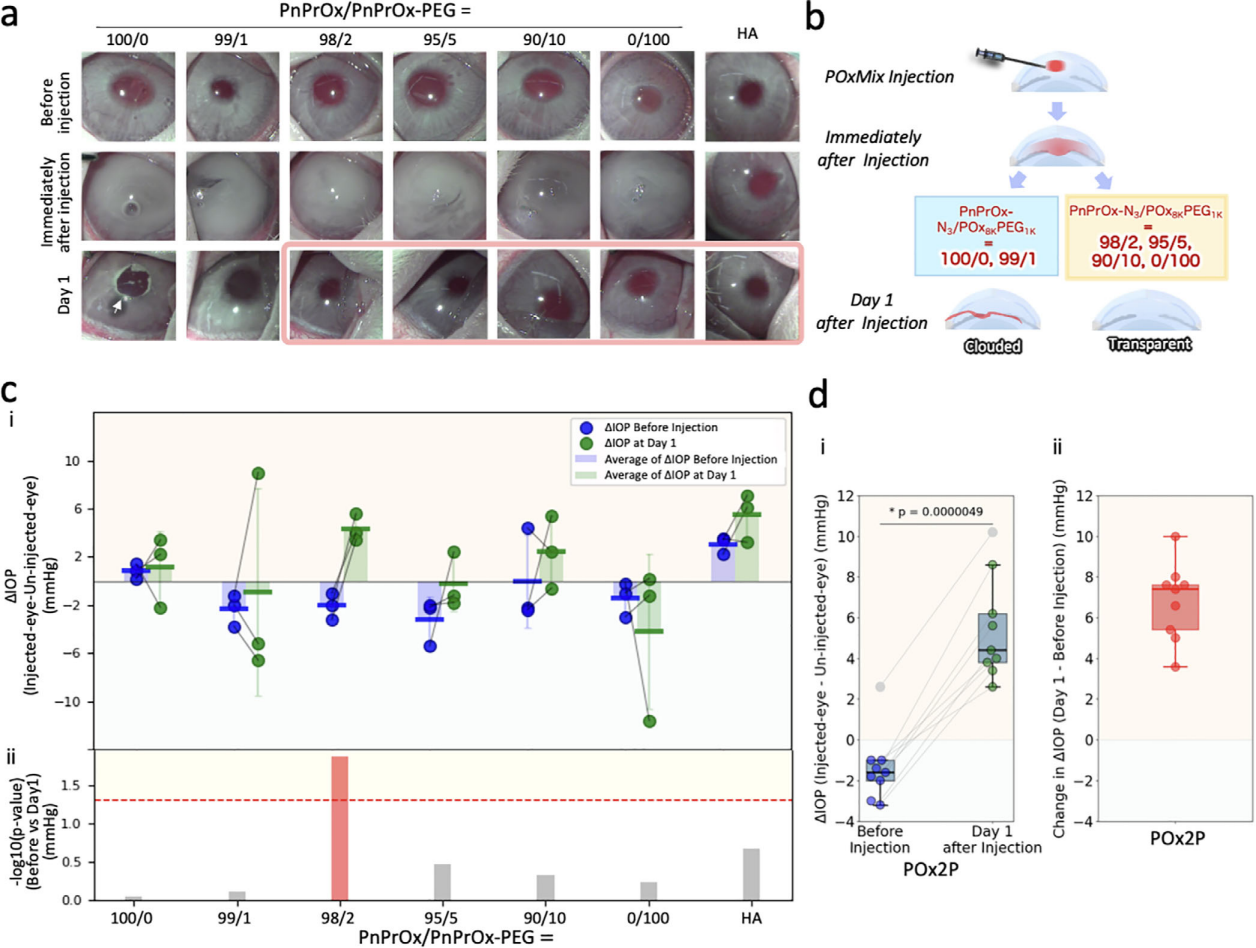
Screening of PnPrOx/PnPrOxPEG mixture solutions for the ability to elevate intraocular pressure (IOP) through anterior chamber injection in a normal rabbit, with clinical‐grade hyaluronic acid (HA) included as a reference control. (a) Anterior segment photographs of PnPrOx/PnPrOxPEG mixture solutions (PnPrOx/PnPrOxPEG = 100/0, 99/1, 98/2, 95/5, 90/10, and 0/100), along with HA solution captured before injection, immediately after injection, and 1 day after injection. Red boxes indicate conditions where anterior segment transparency was maintained. (b) Schematic depicting PnPrOx/PnPrOxPEG mixture solution localization in the anterior chamber after injection in relation to PnPrOx/PnPrOxPEG ratios. (c) Relationship between IOP difference (treated versus untreated eye) 1 day after injection and PnPrOx/PnPrOxPEG mixing ratios, along with HA solution. The panel includes two graphs: (i) ΔIOP values and (ii) p‐value plots from paired statistical testing. In (ii), the red dashed line indicates the threshold of p = 0.05, and data points with p‐values below 0.05 are highlighted with red boxes. (d) Additional validation experiments examining the IOP‐elevating ability of the promising mixing ratio (PnPrOx/PnPrOxPEG = 98/2, *n* = 9). Before injection: mean −1.38 mmHg (95% CI −2.67 to −0.08). Day 1: mean 5.42 mmHg (95% CI 3.48–7.37). ΔΔIOP: mean 6.80 mmHg (95% CI 5.34–8.26). A paired t‐test confirmed a significant increase (p = 4.9 × 10^−^^6^).

To identify a formulation capable of effectively elevating IOP, we screened various PnPrOx/PnPrOxPEG mixtures together with clinical‐grade HA as a reference viscoelastic control, by injecting them into the anterior chamber of normal rabbits and monitoring IOP changes (Figure 2a i). All POx‐based formulations initially demonstrated transient anterior chamber turbidity, whereas HA remained transparent (Figure 2b, middle panel). However, 24 h after injection, only the 100/0 and 99/1 mixtures showed the formation of persistent white precipitates, whereas the 98/2, 95/5, 90/10, and 0/100 mixtures, similar to HA, exhibited complete clearance and restored anterior chamber transparency (Figure 2a, lower panel; Figure 2b).

Among the tested formulations, only the 98/2 mixture, designated POx2P, consistently induced a robust IOP elevation of >5 mmHg in all three tested eyes (Figure 2c (i)). The mean increase in IOP was statistically significant (Figure 2c [ii]). To validate these findings, additional injections of POx2P were performed, confirming a reproducible and significant elevation in ΔIOP at 24 h after injection (Figure 2d (i),(ii)). Specifically, ΔIOP increased from −1.38 mmHg before injection (95% CI −2.67 to −0.08) to 5.42 mmHg at Day 1 (95% CI 3.48–7.37), resulting in a mean ΔΔIOP of 6.80 mmHg (95% CI 5.34–8.26; *n* = 9). We further examined the duration of the IOP‐elevating effect of POx2P and confirmed that the elevated IOP observed on Day 1 returned to the pre‐injection level by Day 2 (Figure S4). Three rabbits were also monitored intermittently for approximately one month as part of welfare follow‐up. Visible observations, consistent with the assessments shown in Figure 2, revealed no abnormalities in the cornea, aqueous humor, or lens. No conjunctival hyperaemia or inflammatory signs were detected, and no delayed elevation of IOP was observed at 3–5 weeks after injection.

### 2.4 Experimental Validation of POx2P in a Rabbit Model of Low IOP

**FIGURE 3:**
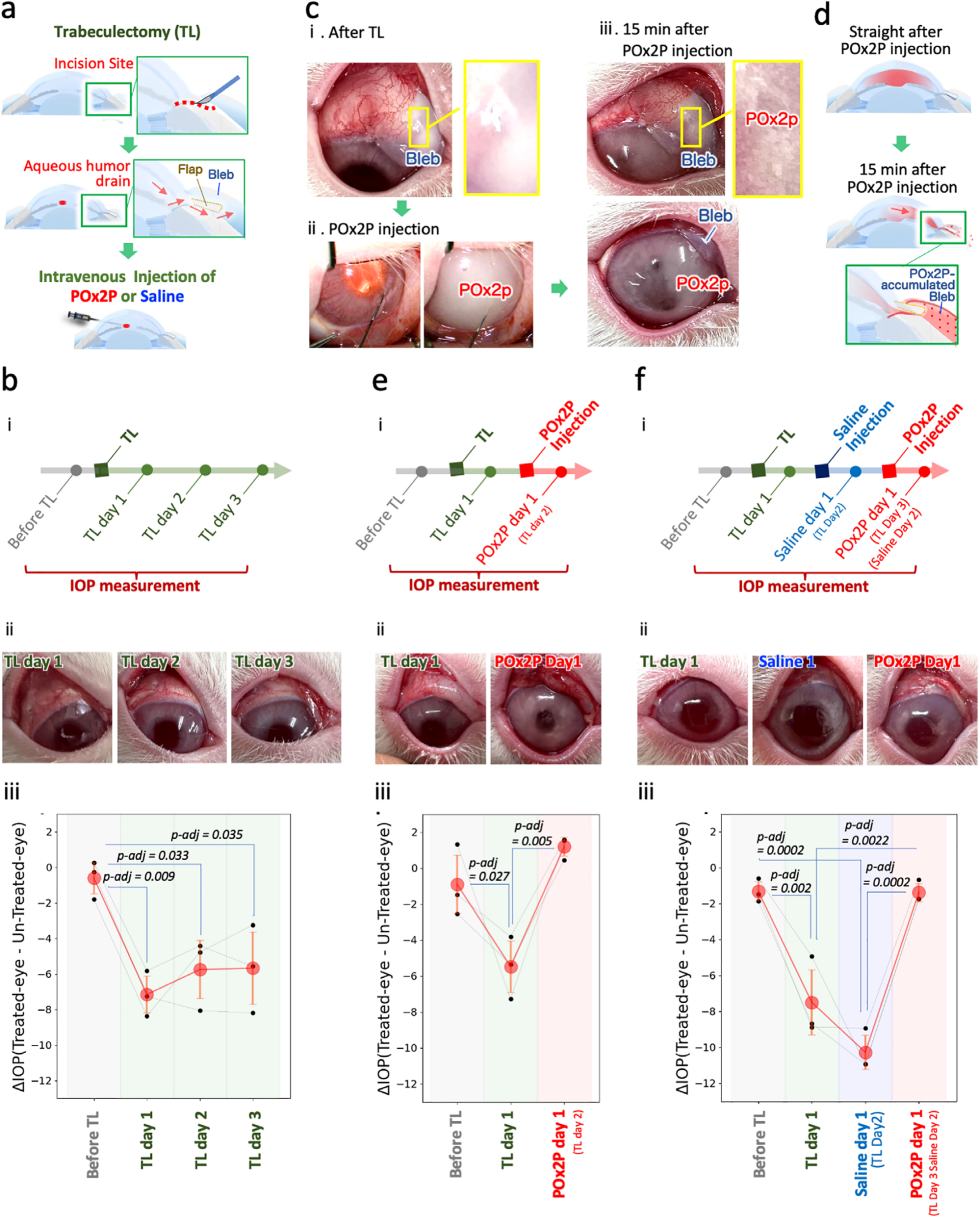
Verification of the IOP‐elevating ability of PnPrOx/PnPrOxPEG mixture solutions (PnPrOx/PnPrOxPEG = 98/2; POx2P) in a rabbit trabeculectomy (TL) model. (a) Overview of TL and injection experiments: TL was performed in one eye of a rabbit to form a conjunctival bleb, followed by anterior chamber injection of POx2P or saline. (b) Temporal changes in IOP differences (treated versus untreated eye) before and after TL: (i) Experimental overview. (ii) Anterior segment images. (iii) IOP measurements. (c) Anterior segment observations with photography: (i) Enlarged images of the anterior segment and bleb post‐TL. (ii) Anterior segment during POx2P injection. (iii) Anterior segment and bleb 15 min after injection. (d) Schematic representation of POx2P localization post‐injection. (e) Temporal changes in IOP differences before and after POx2P injection post‐TL: (i) Experimental overview. (ii) Anterior segment images. (iii) IOP measurements. (f) Temporal changes in IOP differences after saline injection, comparing TL and POx2P injection: (i) Experimental overview. (ii) Anterior segment images. (iii) IOP measurements.

To assess the therapeutic potential of POx2P in a rabbit model of hypotony, TL was performed in one eye of each rabbit (Figure 3a), which led to a sustained IOP reduction of 6–8 mmHg relative to that observed in the untreated contralateral eye (Figure 3b; Figure S5). One day after TL, intraocular injection of POx2P resulted in immediate anterior chamber turbidity (Figure 3c (i),(ii)). Within 15 min, the POx2P solution migrated toward the filtration bleb, forming white aggregates that were absent before the injection (Figure 3c (iii),d).

At 24 h after injection, the IOP, which initially decreased by approximately 5.5 mmHg following TL, was fully restored. Furthermore, the value slightly exceeded that of the contralateral eye by ∼1 mmHg (Figure 3e; Figure S6). In contrast, injection of saline after TL resulted in a further decline in IOP of approximately 2 mmHg (Figure 3f; Figure S7). However, subsequent injection of POx2P after saline administration reversed the decline in IOP and restored it to the levels recorded before TL the following day.

## 3 Discussion

We report a modular strategy that enables the reversible and transparent modulation of IOP through thermally induced aggregation of POx–PEG co‐assemblies. By simply tuning the mixing ratio of two pre‐defined polymers—POx and PEG—we identified a formulation that achieves sustained IOP elevation without compromising anterior chamber clarity. This outcome addresses a key clinical challenge in managing hypotony, where existing interventions often lack durability and may impair optical transparency [11, 12, 13].

The aggregation behavior of POx‐based polymers was highly sensitive to PEG content, with particle size, dispersibility, and fusion characteristics strongly correlated with composition. These results highlight a direct physicochemical link between formulation structure and in vivo performance. Increasing the fraction of POx–PEG sterically shields the hydrophobic PnPrOx domains and reduces inter‐particle adhesion, thereby suppressing fusion and stabilizing particle size. Steric shielding requires PEG chains to be presented directly at the aggregate interface. Therefore, free PEG would not be expected to influence PnPrOx aggregation. This interfacial localization is achieved only when PEG is covalently linked as a POx–PEG block copolymer, enabling composition‐dependent control of aggregate size and dispersibility. Conversely, mixtures with lower PEG content allow stronger PnPrOx–PnPrOx interactions, resulting in larger and more adhesive aggregates. All mixtures were prepared at a fixed polymer concentration (1 mg/mL); higher concentrations would be expected to increase the collision frequency and promote fusion, whereas dilution would suppress aggregation. The reversible clearance observed in vivo is explained by the continuous aqueous humor turnover, where progressive dilution shifts the thermo‐responsive assemblies back toward the dissolved state, leading to gradual size reduction and disappearance. Unlike conventional HA‐based solutions, which are transparent and function primarily by increasing the bulk viscosity [33] yet often lack tunability in retention or outflow resistance and may fail to provide lasting IOP recovery [14, 15, 16], our thermally responsive platform enabled IOP control via compositional modulation of aggregation—a mechanism distinct from those of supramolecular or crosslinked hydrogels. Notably, in our rabbit model, IOP elevation was observed in all tested animals 1 day after injection, highlighting the robustness and reproducibility of this response.

To contextualize the observed 1‐day IOP elevation, we additionally examined intracameral HA. Clinically, a single injection of HA can occasionally restore the anterior chamber depth and promote the long‐term recovery of aqueous production, although several reports [14, 15, 16] have noted that some patients show insufficient IOP normalization. In our experiment, HA elevated IOP in two of three eyes at 24 h, but the effect appeared transient. In contrast, POx2P elevated IOP in all tested eyes, including in the TL model, without signs of overcorrection, suggesting the possibility of a more consistent or controlled response, although this requires further validation and optimization.

Previous polymer systems typically relied on the de novo synthesis of multiple candidates and empirical screening, whereas our two‐component system facilitated rational design and efficient identification of effective formulations through simple compositional adjustment. This is contrary to the complexity of thermogels and supramolecular architectures reported for injectable applications [3, 22, 23, 24]. The COOH–PEG_1_K design was selected as a practical starting point, and because the terminal COOH represents only a negligible fraction of the polymer, major interfacial effects are unlikely. Detailed optimization of end‐groups or PEG length was beyond the scope of the present study.

In vivo validation of the optimized formulation (PnPrOx:PnPrOxPEG = 98:2, POx2P) demonstrated consistent IOP elevation in both healthy rabbits and a trabeculectomy‐induced hypotony model, while maintaining anterior chamber transparency. Microscopy revealed the presence of aggregates near aqueous outflow structures, suggesting that particle size and fusion behavior may transiently modulate outflow resistance, although direct evidence of local deposition has not yet been established. Formulations that exhibited adhesion‐ or fusion‐dominant behavior in vitro (Figure 1f,g) tended to form larger and more persistent aggregates, which aligns with reduced transparency and less stable IOP responses in vivo. In contrast, the 98:2 formulation displayed a more dispersed suspension phenotype (Figure 1h), consistent with the clear anterior chamber and reproducible Day‐1 IOP elevation shown in Figure 2. These parallel trends indicate that the aggregation mode observed in vitro directly influences the in vivo outflow modulation. We employed both histology and a preliminary 37°C flow model to further examine this mechanism. However, the aggregates dissolved during post‐mortem cooling and could not be preserved for pathological analysis, and the simplified flow system did not reproduce the sensitivity of the living eye. Although direct mechanistic proof remains challenging, the reversible IOP changes and imaging findings support the transient modulation of outflow resistance as a plausible explanation. Supporting data further show that IOP returns toward baseline by 48 h, indicating that the effect is transient and reversible.

From a translational perspective, the essential clinical requirement in postoperative hypotony is to rescue the eye from critically low intraocular pressure (<5 mmHg) rather than to elevate pressure into a specific target range. The goal is to restore IOP to a safe level above the hypotonic threshold while preserving anterior chamber and corneal transparency, as visible aggregation would not be clinically acceptable. In this context, POx2P produced a clear yet controlled IOP elevation comparable to or greater than that of intracameral HA, while maintaining full optical clarity, suggesting potential suitability for early‐phase pressure support.

These findings are consistent with previous findings that the physical properties of injected materials influence flow modulation [34, 35], and with work demonstrating compositionally tunable, stimuli‐responsive nanomaterial platforms [36]. Our results extend these concepts to a simplified POx‐based system with translational potential. Multiple techniques have also been developed to enhance the transparency of collagen‐based materials via dehydration and crosslinking, making the structure so uniform that it contains no features large enough to diffract or scatter visible light [37, 38, 39, 40, 41]. Although some of these approaches facilitate injectable transparent gel formation, irreversible gelation throughout the anterior chamber could result in uncontrolled outflow obstruction and excessive IOP elevation. Hence, such strategies were not evaluated in vivo. In contrast, the POx–PEG system did not induce overcorrection even in the hypotony model, highlighting its safety and tuneability.

POx‐based polymers are chemically defined, biocompatible, and metabolically stable [18, 19, 20]. Clinical applications of POx‐based materials are already progressing in haemostasis, oncology, and drug delivery [23, 24, 29]. Our approach—based solely on mixing two existing components—facilitates scalable production and regulatory alignment, offering practical advantages for clinical development. In this study, no abnormalities of the cornea, aqueous humor, lens, or conjunctiva were visible during the entire observation period of the animal experiments.

Despite these promising results, further studies such as nonclinical programs are required to assess long‐term degradation, clearance, and tissue compatibility with histological analysis and performance under variable injection conditions and repeated dosing. Nonetheless, our findings demonstrate that thermally triggered aggregation via compositional tuning of POx–PEG co‐assemblies provides a scalable, transparent, and controllable platform for IOP modulation. This strategy may serve as a broadly applicable foundation for developing flow‐responsive biomaterials for the treatment of pressure‐related ocular conditions and beyond.

## 4 Conclusion

In this study, we explored mixed solutions with varied weight ratios of PnPrOx, a thermo‐responsive polymer, and PnPrOx‐PEG, a block copolymer composed of both thermo‐responsive and hydrophilic segments to obtain particles exhibiting a desirable size and aggregation behavior under physiological temperature, with the overall aim of counteracting decreased IOP. All samples generated microparticles at 37°C. Specifically, when PnPrOx and PnPrOx‐PEG were combined at a weight ratio of 98:2, these particles first underwent rapid Brownian motion, subsequently aggregating and settling as microparticles by 3 min. Injecting this particular solution into the anterior chamber effectively increased the IOP. Conversely, other solutions with varying weight ratios of PnPrOx and PnPrOx‐PEG failed to increase the IOP, which we attribute to factors such as excessively small particle size, suboptimal (either too poor or too high) aggregation behavior, or persistent Brownian motion. Furthermore, this precisely tuned 98:2 particle solution successfully elevated the IOP in eyes that had undergone TL, a common surgical procedure for treating glaucoma. This finding verifies that our material can increase the IOP even in eyes with naturally lower pressure. Considering that hypotony can lead to considerable ocular distress and, in the worst‐case scenario, phthisis bulbi, this developed material shows great promise as an injectable treatment for such eye disorders.

## 5 Methods

### 5.1 Polymer Abbreviation Definitions

In this study, PnPrOx refers to *poly(2‐n‐propyl‐2‐oxazoline)*, a thermo‐responsive POx homopolymer. PnPrOx‐PEG refers to its amphiphilic block copolymer (PnPrOx‐b‐PEG, Mn ≈ 8k‐b‐1k), generated via copper‐free click conjugation of PnPrOx to PEG.

### 5.2 Materials

Super‐dehydrated acetonitrile and sodium azide were purchased from Wako Pure Chemical Co. (Japan). Methyl‐p‐toluene sulphonate (MeTsO), and 2‐n‐propyl‐2‐oxazoline (nPrOx), were purchased from Tokyo Chemical Industry Co. (Tokyo, Japan). α‐dibenzyl cyclooctene (DBCO), and ω‐carboxyl PEG‐COOH (PEG‐COOH) were purchased from Biopharm PEG Scientific Inc. (Watertown, MA).

### 5.3 Synthesis of PnPrOx

PnPrOx was synthesized by employing a previously reported method. Briefly, MeTsO (61.7 mg, 0.33 mmol) was dissolved in acetonitrile (9 mL) and used as an initiator. nPrOx (3.3 g, 29.5 mmol) was added to the initiator solution, and the mixture was stirred at 42°C for 10 days. Polymerization was terminated by adding sodium azide (430 mg, 6.6 mmol); this was followed by stirring at 70°C for 24 h. The reaction mixture was purified by dialysis (MWCO: 3500 Da) against methanol (five times). The sample was concentrated under vacuum and lyophilized using 1,4‐dioxane to obtain a white powder.

### 5.4 Synthesis of PnPrOx‐PEG

PnPrOx (400 mg, 0.05 mmol) and DBCO‐PEG‐COOH (100 mg, 0.5 mmol) were dissolved in 6 mL of methanol and stirred at 40°C for 2 days. The reaction mixture was purified by dialysis (MWCO: 3500 Da) against methanol (five times). The resulting sample was concentrated under vacuum and lyophilized using 1,4‐dioxane to obtain a yellow powder. The obtained polymer was characterized using ^1^H NMR spectroscopy.

### 5.5 Thermo‐Responsivity of PnPrOx/PnPrOxPEG Mixtures

PnPrOx and PnPrOx‐PEG were dissolved in PBS at a concentration of 1 mg/mL. The transmittance of 500 nm light through PnPrOx, PnPrOx‐PEG, and their 9:1 mixture was assessed using UV–vis spectroscopy (V‐650, JASCO Co., Tokyo, Japan). The temperature was increased at a rate of 1°C/min from 20°C to 50°C.

### 5.6 Microscopy‐Based Imaging of Thermo‐Responsive Phase Transition

Polymer solutions and mixtures were placed in a disc‐shaped chamber (diameter: 8 mm; silicon rubber thickness: 100 µm), sandwiched between cover glasses, and fixed on a transparent glass heater at 37°C. Observations were performed using an optical microscope (BZ‐X710, Keyence, Kyoto, Japan).

To confirm the phase transition, the glass heater was cooled with ice water for 20 s, after which the ice water was removed. The gradual precipitation process was recorded using a time‐lapse module (BZ‐H3XT, Keyence). Particle size distributions at 0, 3, and 5 min after ice removal were analyzed using ImageJ (U.S. National Institutes of Health, Bethesda, MD, USA).

### 5.7 Animal Experiments

All animal experiments were performed in adherence with the ARVO Statement for the Use of Animals in Ophthalmic and Vision Research and were approved by the Experimental Animal Management Committees of Yamanashi University (approval number: A1‐1), Tsukuba University (approval number: 23–434), and Tohoku University (approval number: 2024‐001‐02). Male Japanese White rabbits (20 weeks or older; Kitayama Labs, Japan) were used.

Before intravitreal injection or trabeculectomy, the anterior segment was examined to confirm the absence of abnormalities. IOP was measured using a retentive device in the awake state, and only rabbits with an IOP between 10 and 30 mmHg in both eyes were included.

The experimental design is described as follows: To screen for IOP‐elevating effects, various PnPrOx/PnPrOx‐PEG mixtures were injected into normal rabbit eyes to assess their effects on IOP. To validate IOP‐elevating effects in a low‐IOP model, the most effective test substances identified during the screening were injected into rabbit eyes after trabeculectomy‐induced IOP reduction.

### 5.8 Injection Protocol

In Experiment (1), multiple injections were administered to the same rabbit, spaced at least one month apart. IOP was confirmed to be within the 10–30 mmHg range before each injection, and different test materials were used for each injection to avoid repeated exposure.

In Experiment (2) (trabeculectomy model), a new rabbit was used for each procedure.

### 5.9 Anaesthesia Protocol

Before surgery, the animals were intramuscularly anaesthetized with ketamine hydrochloride (60 mg k^−1^g; Sankyo, Tokyo, Japan) and xylazine (10 mg k^−1^g; Bayer, Munich, Germany) as described in previous literature [37].

### 5.10 Injection of Solutions into the Anterior Chamber

IOP was measured in awake rabbits before injection. The anterior segment was photographed, and the rabbits were anaesthetized prior to injection. For anterior chamber injection, approximately 100 µL of aqueous humor was withdrawn via the cornea using a 32 G needle and a 1 mL syringe to prevent IOP overload. A total of 100 µL of the PnPrOx/PnPrOx‐PEG mixture solution (PnPrOx/PnPrOxPEG = 100/0, 99/1, 98/2, 95/5, 90/10, and 0/100) dissolved in saline at a concentration of 1 mg/mL, or saline alone as control, was injected using the same 32 G needle and 1 mL syringe. Images were captured immediately after the injection. IOP was re‐evaluated 1 day later under awake conditions.

### 5.11 Trabeculectomy in Rabbits

Trabeculectomies were performed by a single surgeon (SM). The right eye was designated as the operative eye. The rabbits received intravenous sodium pentobarbital (30 mg k^−1^g) prior to surgery. The third eyelid was excised to prevent surgical field obstruction. The conjunctiva and Tenon's capsule were opened using a limbal‐based approach using micro‐spring scissors.

A half‐thickness scleral flap (3 × 3 mm) was dissected until the trabeculae were visible. A 2 mm × 1 mm trabeculectomy was performed using a 15° or 30° blade, followed by peripheral iridectomy. The scleral flap was left unsutured, and the conjunctiva was closed using 10‐0 nylon sutures (Mani, Utsunomiya City, Japan). At the end of the procedure, 0.3% ofloxacin ophthalmic ointment was applied.

### 5.12 IOP Measurement

IOP was measured under awake conditions using a TonoVet handheld tonometer equipped with a holding device. To minimize fluctuations, the following steps were performed:

Five consecutive readings (25 knocks per eye) were captured. This process was repeated thrice. An average of 75 readings per eye was used as the final IOP. Since left and right IOP varied among individuals, the difference between the treated and untreated eyes (ΔIOP) was calculated. The IOP before and after treatment was compared to evaluate the effect of IOP elevation.

### 5.13 Statistical Analysis

Paired t‐tests were performed to evaluate the ability of PnPrOx/PnPrOx‐PEG mixtures before and after intraocular injection. In the rabbit TL model, analysis of variance (ANOVA) was conducted to compare data before TL, after TL, and 1 day after drug administration (mixture or saline).

## Author Contributions

Y.T. conceived the concept of IOP elevation using PnPrOx, initiated the design of a PnPrOx/PnPrOx‐PEG blend library and in vivo screening in rabbits, optimized the library formulation, established and performed microscopic observation techniques, developed the injection and IOP measurement protocols in rabbits, conducted the animal experiments, secured funding, analyzed the data, and wrote the manuscript. S.O. designed, synthesized, characterized and purified the PnPrOx and PnPrOx‐PEG polymers and contributed to manuscript preparation. S.M. performed the trabeculectomy procedures and contributed to manuscript writing. All authors reviewed and approved the final version of the manuscript.

## Funding

This research was supported by JSPS KAKENHI (Challenging Research, 18K19948), the Kobe Foundation, and AMED's Translational Research Program (Seeds A A19‐56, preF 19–56, and A26‐16).

## Conflicts of Interest

The authors declare no conflicts of interest.

## Supporting information




**Supporting File 1**: adhm71132‐sup‐0001‐SuppMat.docx.

**Supporting File 2**: adhm71132‐sup‐0002‐MovieS1.mp4.

**Supporting File 3**: adhm71132‐sup‐0003‐MovieS2.mp4.

**Supporting File 4**: adhm71132‐sup‐0004‐MovieS3.mp4.

**Supporting File 5**: adhm71132‐sup‐0005‐MovieS4.mp4.

**Supporting File 6**: adhm71132‐sup‐0006‐MovieS5.mp4.

**Supporting File 7**: adhm71132‐sup‐0007‐MovieS6.mp4.

## Data Availability

The data that support the findings of this study are available from the corresponding author upon reasonable request.
