# Supplementary material for: Modular Control of PEG‐Poly(2‐Oxazoline) Co‐Assembly Enables Tunable Aggregate Properties for Intraocular Pressure Management
Source: Adv Healthc Mater. 2026 Apr 9;15(30):e04206. doi: 10.1002/adhm.202504206 (PMC13474139; doi:10.1002/adhm.202504206)
Supplement: Supplementary file 1 [file ADHM-15-0-s002.docx]

Supporting Information

Modular Control of Poly(2-Oxazoline) Aggregation via PEG Co-assembly Enables Tuneable Thermo/Flow-Responsive Intraocular Pressure Management

Yuji Tanaka^1,2*^, Shohei Morikawa^~~2~~3^**^~~†~~^**, and Shigeto Osawa^~~3~~4^**^~~†~~^**

^1*^New Industry Creation Hachery Center, Tohoku University, 6-6-10, Aramaki, Aoba-ku, Sendai, Miyagi, 980-8579, Japan

^2^Innovation Center of NanoMedicine (iCONM), Kawasaki Institute of Industrial Promotion, 3-25-14 Tonomachi, Kawasaki-ku, Kawasaki 210-0821, Japan

^~~2~~3^Faculty of medicine, University of Tsukuba, 1-1-1 Tennodai, Tsukuba, Ibaraki 305-8577, Japan

^~~3~~4^Department of Biomedical Engineering, Faculty of Life Science, Toyo University, 48-1 Oka, Asaka, Saitama 351-8510, Japan

((Please insert your Supporting Information text/figures here. Please note: Supporting Display items, should be referred to as Figure S1, Equation S2, etc., in the main text…)

**Supplementary Figure 1. Synthetic scheme of PnPrOx (a) and PnPrOx-*b*-PEG (b).**

**Supplementary Figure 2. ^1^H-NMR spectra of the obtained PnPrOx-*b*-PEG.**

**Supplementary Figure 3. SEC chromatogram of PnPrOx-PEG.**

**Supplementary Figure 4. Sustained intraocular pressure–elevating effect of POx2P following intracameral injection in normal rabbits.** (a) Experimental overview:Normal rabbit eyes received an intracameral injection of the POx2P formulation (PnPrOx-N3/POx8KPEG1K-XX = 98/2). Intraocular pressure (IOP) was measured immediately before injection (Before injection), 1 day after injection (Day 1), and 2 days after injection (Day 2). (b) Time course of ΔIOP (treated eye − untreated eye): The ΔIOP values at each time point are presented. A marked elevation in IOP was observed on Day 1, followed by a decline toward baseline on Day 2. (c) Statistical analysis: Paired t-tests were performed to compare matched measurements across time points (N = 3). (i) Before injection vs Day 1, (ii) Before injection vs Day 2, (iii) Day 1 vs Day2.

**Supplementary Figure 5: Statistical evaluation of intraocular pressure (IOP) changes before and after trabeculectomy (TL) in rabbits.**
IOP differences between treated and untreated eyes (ΔIOP) were analysed across four conditions: before TL, TL-day-1, TL-day-2, and TL-day-3. One-way ANOVA revealed a significant effect of condition on ΔIOP (F = 7.65, *p* = 0.0098). Post-hoc Tukey’s HSD tests indicated that ΔIOP at TL-day-1 was significantly greater than that before TL (mean difference = 6.55 mmHg, *p* = 0.0092). Similarly, the ΔIOP was significantly higher at TL-day-2 and TL-day-3 compared to the pre-TL baseline (*p* = 0.0329 and *p* = 0.0354, respectively). No significant differences were observed among the post-TL days (e.g., TL-day-1 vs. TL-day-2), suggesting a comparable effect of POx2P injection on IOP restoration across the observed time points. To complement these findings, a non-parametric Kruskal–Wallis test was performed, which yielded a borderline result (H = 7.21, *p* = 0.0656). Although not significant at the conventional 5% level, this result suggests a trend consistent with the ANOVA-based group differences. Taken together, these results support the interpretation that POx2P injection led to a significant restoration or elevation of IOP following TL surgery, relative to the pre-injection condition.

**Supplementary Figure 6: Statistical evaluation of intraocular pressure (IOP) recovery following POx2P injection after trabeculectomy (TL) in rabbits.**
IOP differences (ΔIOP) between treated and untreated eyes were compared across three conditions: before TL, TL-day-1 (1 day after trabeculectomy), and POx2P-day-1 (1 day after POx2P injection). One-way ANOVA revealed a significant effect of condition on ΔIOP (F = 14.12, *p* = 0.0054). Tukey’s HSD post-hoc analysis showed that the ΔIOP at POx2P-day-1 was significantly lower than that at TL-day-1 (mean difference = −6.67 mmHg, *p* = 0.0049), indicating effective IOP recovery. However, no significant difference was observed between POx2P-day-1 and before TL (*p* = 0.3056), suggesting a near-complete restoration of the IOP to baseline levels. A significant difference was also observed between TL-day-1 and before TL (*p* = 0.0274), confirming the persistence of hypotony prior to POx2P treatment. To validate these results, a Kruskal–Wallis test was conducted, which revealed a significant group effect (H = 6.49, *p* = 0.0390), consistent with the findings of the ANOVA. Collectively, these results demonstrate that POx2P injection significantly reversed TL-induced IOP reduction within 24 h, restoring pressure to near pre-surgical levels.

**Supplementary Figure 7: Statistical evaluation of intraocular pressure (IOP) dynamics following saline and POx2P injections in the trabeculectomy (TL) model.**
To assess the effect of sequential treatments, differences in IOP (ΔIOP) were compared across four conditions: before TL, TL-day-1 (post-surgery), Saline-day-1 (TL-day-2; 1 day after saline injection), and POx2P-day-1 (TL-day-3; 1 day after subsequent POx2P injection). One-way ANOVA revealed a highly significant effect of condition on ΔIOP (F = 34.44, *p* < 0.0001). Tukey’s HSD post-hoc test showed that the ΔIOP at POx2P-day-1 was significantly lower than that at both TL-day-1 (mean difference = −6.11 mmHg, *p* = 0.0022) and Saline-day-1 (–8.89 mmHg, *p* = 0.0002), indicating effective restoration of the IOP following POx2P administration. In contrast, Saline-day-1 exhibited significantly greater ΔIOP than before TL (*p* = 0.0002), suggesting continued hypotony. No significant difference was observed between POx2P-day-1 and the pre-TL baseline (*p* = 0.9999), indicating that IOP was restored to near-baseline levels only after POx2P injection. A Kruskal–Wallis test confirmed a significant group effect (H = 9.42, *p* = 0.0241), further supporting the robustness of these findings. Overall, these results demonstrate that saline injection alone did not reverse TL-induced hypotony, whereas subsequent POx2P injection restored the IOP to pre-surgical levels.

**Supplementary Movie 1: Time-lapse phase-contrast microscopy of thermally triggered aggregation of PnPrOx-N₃ in phosphate-buffered saline (PBS).**
A PnPrOx-N₃ solution (1 mg/mL in PBS) was pre-incubated for over 5 min in a sealed observation chamber on a phase-contrast microscope stage maintained at 37 °C. Aggregates were confirmed to be present before imaging. Approximately 10 s after the start of recording, an ice pack was gently placed on top of the chamber for 30 s without changing the stage heater setting (37 °C), leading to a transient temperature drop. During this period, previously visible aggregates fully dissolved. Upon removal of the ice, reaggregation was observed: small particles gradually formed, grew in size, and adhered to the glass substrate. Collisions and fusion among particles continued during the first few minutes, after which no further morphological changes were observed. The entire process was captured over a >5-min time-lapse sequence.

**Supplementary Movie 2: Time-lapse phase-contrast microscopy of thermally triggered aggregation of PnPrOx-N₃/POx8KPEG1K (99/1) mixture in phosphate-buffered saline (PBS).**
A 99:1 (w/w) mixture of PnPrOx-N₃ and POx8KPEG1K was prepared in PBS at a concentration of 1 mg/mL and sealed in a chamber on a phase-contrast microscope stage maintained at 37 °C. After confirming the presence of aggregates following a 5-min pre-incubation, recording was initiated. At approximately 15 s, an ice pack was applied to the chamber for 30 s, without altering the stage temperature, causing transient cooling and complete dissolution of visible aggregates. Upon removal of the ice, reaggregation was observed. Compared to the 100/0 formulation (Supplementary Movie 1), the newly formed aggregates were smaller in size. All particles settled onto the glass substrate and adhered without exhibiting any Brownian motion. Particle growth and fusion progressed over several minutes and reached a stable morphology within approximately 5 min.

**Supplementary Movie 3:** **Time-lapse phase-contrast microscopy of thermally triggered aggregation of PnPrOx-N₃/POx8KPEG1K (98/2) mixture in phosphate-buffered saline (PBS).**
A 98:2 (w/w) mixture of PnPrOx-N₃ and POx8KPEG1K was dissolved in PBS (1 mg/mL) and pre-incubated at 37 °C for over 5 min in a sealed microscopy chamber. After confirming aggregate formation, time-lapse recording was initiated. Ten seconds into recording, an ice pack was applied to the chamber for 30 s (with stage temperature maintained at 37 °C), causing transient cooling and complete dissolution of aggregates. Upon removal of the ice, reaggregation began, forming smaller particles compared to those in the 99/1 mixture (Supplementary Movie 2). Most particles gradually grew and adhered to the glass surface, where they remained immobile. However, a small number of smaller particles remained suspended and exhibited persistent Brownian motion throughout the 5-min observation period. This indicates partial modulation of particle–surface interactions by the increased PEG content.

**Supplementary Movie 4:** **Time-lapse phase-contrast microscopy of thermally triggered aggregation of PnPrOx-N₃/POx8KPEG1K (95/5) mixture in phosphate-buffered saline (PBS).**
A 95:5 (w/w) mixture of PnPrOx-N₃ and POx8KPEG1K was dissolved in PBS at a concentration of 1 mg/mL and incubated at 37 °C in a sealed microscopy chamber for over 5 min prior to imaging. After confirming the presence of aggregates, time-lapse recording was initiated. At approximately 10 s, an ice pack was placed on top of the chamber for 30 s, inducing transient cooling without changing the stage heater setting. This led to complete dissolution of aggregates. Following removal of the ice, reaggregation was observed. Compared to the 98/2 mixture (Supplementary Movie 3), more particles remained suspended and exhibited persistent Brownian motion, whereas others adhered to the glass substrate and gradually fused over time. The presence of both mobile and immobile particles suggests an increased degree of colloidal stability and reduced surface adhesion due to the higher PEG content. Particle behaviour stabilised within approximately 5 min.

**Supplementary Movie 5:** **Time-lapse phase-contrast microscopy of thermally triggered aggregation of PnPrOx-N₃/POx8KPEG1K (90/10) mixture in phosphate-buffered saline (PBS).**
A 90:10 (w/w) mixture of PnPrOx-N₃ and POx8KPEG1K was prepared in PBS (1 mg/mL) and pre-incubated at 37 °C for over 5 min in a sealed chamber mounted on a phase-contrast microscope. Following confirmation of aggregate formation, time-lapse recording was initiated. At approximately 15 s, an ice pack was placed on the chamber for 30 s without changing the stage temperature, resulting in transient cooling and full dissolution of aggregates. Upon removal of the ice, particle reaggregation occurred. Unlike the lower PEG ratio conditions (e.g., 95/5, Supplementary Movie 4), all reformed particles remained suspended throughout the >5-min observation period and exhibited persistent Brownian motion without adhering to the glass surface. No particle fusion or size increase was observed. These findings indicate that the higher PEG content significantly inhibits particle–surface interactions and stabilises dispersed micro-aggregates under physiological temperature.

**Supplementary Movie 6:** **Time-lapse phase-contrast microscopy of thermally triggered aggregation of POx8KPEG1K alone (0/100) in phosphate-buffered saline (PBS).**
A solution containing POx8KPEG1K alone (0:100 w/w ratio of PnPrOx-N₃ to POx8KPEG1K) was prepared in PBS (1 mg/mL) and pre-incubated at 37 °C for over 5 min in a sealed chamber on a phase-contrast microscope stage. Following confirmation of temperature-dependent behaviour, time-lapse recording was initiated. At approximately 15 s, an ice pack was applied to the chamber for 30 s while maintaining the stage temperature, resulting in transient cooling and disappearance of scattered particles. Upon removal of the ice, numerous small particles reappeared and remained suspended throughout the >5-min observation period, exhibiting persistent Brownian motion without any adhesion to the glass substrate. Compared to the 90/10 mixture (Supplementary Movie 5), the particles were markedly smaller and showed no evidence of fusion or settling. These findings suggest that POx8KPEG1K alone forms highly stable, submicron-scale aggregates with minimal surface interaction under physiological conditions.
